# Texas State Medical Association

**Published:** 1889-04

**Authors:** 


### TEXAS STATE MEDICAL ASSOCIATION.

### OFFICERS OF SECTIONS, ETC.

For convenience of those who desire to contribute papers, we reproduce the list of chairmen of the several sections—and also of committees:

Practice of Medicine and Materia Medica—Dr. R. B. White, Ennis, chairman; Dr. R. A. McCall, Ennis, secretary.

Obstetrics and Diseases of Children—Dr. W. H. Wilkes, Waco, chairman.

Surgery and Anatomy—Dr. J. C. Jones, Gonzales, chairman.

State Medicine and Public Hygiene—Dr. R. M. Swearingen, Austin, chairman.

Gynecology—Dr. B. E. Hadra, Galveston, chairman; Dr. Geo. H. Lee, secretary.

Medical Jurisprudence, Psychology and Chemistry—Dr. D. R. Wallace, Terrell, chairman.

Ophthalmology and Otology—Dr. R. H. Chilton, Dallas, chairman.

Electro Therapeutics– Dr. W. M. Powell, Albany, chairman.

Dermatology—Dr. H. L. Taylor, Waco, chairman.

Committee of Arrangements—Dr. F. Herff, San Antonio, chairman.

The following committees are expected to report:

Collection Surgical Cases—Dr. Geo. Cupples, chairman.

Legislation—Dr. Geo. Cupples. chairman.

[Necrology—no appointment made.]

[Prize Essay—no appointment made.]

Committee to whom was referred the new draft of Constitution and By-Laws presented at last meeting and not disposed of—Dr. R. H. Harrison, sr., chairman.

Committee to examine paper selected by Prize Essay committee for presentation to the Association, and recommended for the prize—Dr. G. P. Hall, chairman.

Committee to memorialize the Legislature to set apart a room in the Capitol for the Association—Dr. F. R. Martin, chairman.

The Judicial Council consists of Drs. W. L. York, Decatur; J. R. Johnson, Bryan; H. H. Thorpe, Liberty Hill; D. M. Ray, Whiteright. P. C. Coleman, Colorado; R. Rutherford, Houston; M. D. Knox, Hillsboro; R. C. Nettles; Marlin; I. E. Clarke, Schulenburg; C. M. Ramsdell, Lampasas; T. J. Bell, Tyler; R. H. Harrison, sr., Columbus.

---

NOTICE TO MEMBERS TEXAS STATE MEDICAL ASSOCIATION.—It takes money to conduct the business of the Association. Two successive years the Publishing Committee have been in straits for means to issue the transactions in good form; many are shamefully delinquent, and *must pay* up. Come to San Antonio prepared to pay in full. Every member will be required to pay, upon registering; that is the rule, and will be enforced. Those who desire to remit upon receipt of this, and before the meeting, as well as those who will not attend, are requested to send the money to the Treasurer, Dr. J. Larenden at Houston, and *not to the Secretary*.

---

PLEASANT FOR UNCLE JACK.—(Uncle Jack returns from a long walk, and, being somewhat thirsty, drinks from a tumbler he finds on the table. Enter his little niece Allie, who instantly sets up a yell of despair.)

Uncle Jack: "What's the matter, Allie?"

Allie (weeping): "You've drinked up my aquarium and swallowed my free 'ittle pollywogs."

---

DRS. L. B. CREATH, of Kinneyville, and Q. A. Shuford, of Tyler, are attending a special course at the N. O. Polyclinic.

